# Temper outbursts in Lowe syndrome: Characteristics, sequence, environmental context and comparison to Prader–Willi syndrome

**DOI:** 10.1111/jar.12613

**Published:** 2019-05-29

**Authors:** Helen Cressey, Chris Oliver, Hayley Crawford, Jane Waite

**Affiliations:** ^1^ The Cerebra Centre for Neurodevelopmental Disorders, School of Psychology University of Birmingham Birmingham UK; ^2^ Faculty of Health and Life Sciences Coventry University Coventry UK; ^3^ School of Life and Health Sciences Aston University Birmingham UK

**Keywords:** behavioural phenotypes, challenging behaviours, intellectual disabilities, Lowe syndrome, temper outbursts

## Abstract

**Background:**

There is limited research into the nature and aetiology of temper outbursts in people with intellectual disabilities. In this study, we describe the phenomenology and environmental context of temper outbursts in Lowe syndrome, a rare genetic syndrome in which outbursts are purportedly frequent.

**Method:**

A temper outburst interview (TOI) was conducted with caregivers of seventeen individuals with Lowe syndrome to generate an account of the behavioural sequence, common antecedents and consequences of temper outbursts, and to enable comparisons with similar work on Prader–Willi syndrome.

**Results:**

Outbursts in Lowe syndrome were frequently triggered by thwarted goal‐directed behaviour and were associated with high levels of physical aggression and property destruction.

**Conclusions:**

Form and sequence of outbursts showed similarities to Prader–Willi syndrome and to behaviours reported in literature on typically developing children. The results highlight the importance of considering shared aetiology as well as syndrome‐specific pathways in the development of outbursts.

## 1 INTRODUCTION

Temper outbursts are typically included under the rubric of challenging behaviour in intellectual disability research alongside behaviours such as self‐injury and aggression. The prevalence of temper outbursts in people with intellectual disabilities is high, ranging from 24.9% to 34.9% (Smith, Branford, Collacott, Cooper, & McGrother, 1996). Among those with intellectual disabilities and challenging behaviour, outbursts have been reported in 85% of adults and 74% of children (Lowe et al., 2007). However, there have been few studies which examine this phenomenon in detail in an intellectual disability population. This is important as, arguably, some features of temper outbursts may not be considered as operant (learned) behaviours but may reflect other factors involved in the regulation of emotion. In this study, we describe temper outbursts in Lowe syndrome (LS), a rare chromosomal disorder in which atypically high levels of temper outbursts have been reported (Arron, Oliver, Moss, Berg, & Burbidge, 2011; Dolinsky, Jacobs, & Knight, 2008; Kenworthy, Park, & Charnas, 1993). The study complements previous work on Prader–Willi syndrome (PWS) (Tunnicliffe, Woodcock, Bull, Oliver, & Penhallow, 2014) and affords the opportunity to further develop understanding of this phenomenon across syndromes.

Lowe syndrome (oculocerebrorenal syndrome) affects mostly males, with an estimated prevalence of 1 in 500,000 (Loi, 2006). The syndrome is caused by a mutation of the OCRL1 gene, located at Xq26.1 (Yuksel, Karaca, & Albayram, 2009), impacting the development of the eyes, brain, and kidneys (Lewis, Nussbaum, & Brewer, 2012).  Intellectual disability is common (10%–25% mild‐borderline; 25% mild‐moderate; 50%–65% severe to profound; Lewis et al., 2012). To date, a small number of studies have described the behavioural characteristics of LS, with temper outbursts reported in 96% of a sample of 47 male participants (Kenworthy et al., 1993).

Definitions of temper outbursts in intellectual disabilities are varied and vague (Tunnicliffe, 2012). In the typical development literature, temper outbursts are described via constituent behaviours including crying, whining, yelling or shouting, screaming, hitting, kicking, stiffening body, pushing/pulling/grabbing, throwing objects and running away (Potegal & Davidson, 2003). Although there is little consensus on specific behaviours which constitute an outburst there is some agreement that an outburst consists of a cluster of behaviours, critically including an emotional component, and cannot be defined by one behaviour alone.

The majority of the literature on challenging behaviour in intellectual disabilities has adopted a functional analytic approach based on operant learning theory (Emerson, 1993) which asserts that behaviours are maintained by positive and negative social, and automatic reinforcement. For example, an attempt to calm or soothe a child by providing attention or distraction with tangible items may reward behaviours. The child is then more likely to repeat these behaviours when in the same situation in the future (Carr & Durand, 1985). There is a strong evidence base for the functional analytic approach (e.g., Beavers, Iwata, & Lerman, 2013) but an exclusively operant learning approach cannot adequately explain why some challenging behaviours are demonstrated more frequently by people with particular genetic syndromes (Arron et al., 2011; Waite et al., 2014). Temper outbursts are more prevalent in several genetic syndromes, including PWS, Cri‐du‐Chat, Smith‐Magenis (Dykens, Hodapp, & Finucane, 2000) and LS (Kenworthy et al., 1993). This partial specificity (see Dykens et al., 2000) is difficult to explain either from an exclusively biological or operant conditioning perspective. A perspective that incorporates an interaction between the biological/developmental consequences of genetic difference and environmental factors is needed (Tunnicliffe & Oliver, 2011).

Peak prevalence of temper outbursts in typical development lies between 2 and 5 years (Bhatia et al., 1990; Potegal & Davidson, 2003), which coincides with developments in executive function as well as communication and social skills. Outbursts have also been noted in a mixed population of typically developing and intellectual disability older children (aged 5–12 years) referred for inpatient psychiatric treatment (Carlson, Potegal, Margulies, Gutkovich, & Basile, 2009). In this population, outbursts were referred to as “rages” or “angry‐agitated outbursts” and understood as an impairment of self‐regulatory executive function mechanisms.

Evidence is emerging for the potential importance of cognitive deficits as an explanation for behavioural phenotypes (Tunnicliffe & Oliver, 2011). For example, recent work by Woodcock, Humphreys, Oliver, and Hansen (2010) using functional magnetic resonance imaging (fMRI) techniques has linked temper outbursts in PWS to cognitive impairments related to task switching. If temper outbursts in LS are characterized by similar patterns of behaviour to the outbursts in PWS, there may be a common impairment of executive function which interacts with environmental contingencies. This paper provides a descriptive comparison of outburst behaviours in LS and PWS, laying the foundations of potential future research on the links between executive function and outbursts in LS.

This study seeks to increase understanding of common antecedents and behavioural sequence in temper outbursts in LS to inform future studies on the aetiology of outbursts in people with intellectual disabilities and contribute to development of effective intervention strategies. It applies methods from Tunnicliffe et al., (2014), who described temper outbursts in PWS. By adopting this “bottom‐up” descriptive approach, this study will be able to identify both emotional characteristics and typically operant behaviours that comprise the temper outbursts. The replication of Tunnicliffe et al.’s interview methodology also allows for direct comparisons to be made between the two papers.

## 2 METHOD

### 2.1 Ethical approval

Ethical approval was provided by the NHS Research Ethics Committee (Wales‐REC‐4), and written consent obtained from all informants. Pictorial consent forms were used as part of a wider LS study to explain the purpose of the research to people with LS and where possible, to gain direct consent from them to talk to caregivers about their daily lives.

### 2.2 Recruitment

Primary caregivers of 17 people with LS were recruited via an existing study being conducted into the behavioural phenotype of LS, with support from the Lowe Syndrome Trust in the United Kingdom and the Lowe Syndrome Association in the United States. Seven participants were recruited from an existing database held by the research institution.

### 2.3 Participants

Primary carers (informants) were fourteen mothers, one adoptive mother and three fathers, with one couple interviewed together.  The people they cared for (participants) were male, had LS diagnosed by a paediatrician, ophthalmologist or geneticist and were aged between eight and 37 years (*M = *18.29 years*; n* = 9, under 18 years; *n = *8, 18 years or over). Eleven resided in the USA, five in the UK and one in Australia. Adaptive functioning and developmental age were measured using the Vineland Adaptive Behavior Scale—version II (VABS‐II; Sparrow, Cicchetti, Balla, & Doll, 2005) (see Table 1).  Developmental age, (calculated as an age equivalent score using the average of 11 VABS‐II subscale scores) ranged from 0 years and 10 months to 10 years and 8 months (*M = *4 years and 6 months*; n* = 11, less than five years; *n = *5, 5 years or more). All were living in the family home with the informant, except for one who had died six months prior to the interview and had lived part‐time with the informant. The interview schedule was adapted for this informant, for example, asking them to describe temper outbursts in the last month of their son's life rather than the last calendar month.

**Table 1:** Demographic information and adaptive behaviour scores for participants

Part. Ref.ᵇ	Age (y)	Additional diagnosis	Adaptive behaviour: standard scoresª					
			Commᵇ	DLSᵇ	Socialᵇ	Motor	ABCᵇ	AEᵇ
1	8	–	65	66	66	67	65	3:3
2	8	–	70	68	80	67	71	4:3
3	8	–	90	76	85	67	82	4:11
4	9	Haemophilia	62	58	57	56	60	2:6
5	9	–	62	62	62	64	62	3:3
6	9	–	70	68	76	61	70	4:6
7	12	–	72	59	64	64	64	5:3
8	15	–	45	30	48	56	39	2:2
9	17	ASD	35	28	43	51	32	1:8
10	19	–	26	25	32	40	23	0:10
11	20	–	75	71	80	81	83	10:8
12	21	Arthritis	69	63	89	70	72	10:6
13	25	OCDᵇ, ASD	48	52	43	72	47	8:11
14	28	ASDᵇ	21	29	52	44	31	5:11
15	30	–	21	21	20	22	20	1:9
16	36	–	21	21	20	22	20	0:10
17	37	–	naᶜ	na	na	na	na	–

Note

All participants were male.

^ª^ Standard scores from VABS‐II (Sparrow et al., 2005). Standard scores represent level of functioning and correspond to the following categories: high: 130+; moderate high: 115–129; adequate: 86–114; moderate low: 71–85; low: 70 and below.

^ᵇ^ ABC, adaptive behaviour composite; AE, age equivalent score in years: months, calculated as an average of 11 VABS subscale scores; ASD, autism spectrum disorder; Comm, communication; DLS, daily living skills; OCD, obsessive compulsive disorder; Part. Ref., participant reference; Social, socialization.

^ᶜ^ na, not available.

### 2.4 Procedure

Semi‐structured interviews were conducted by the same researcher, by telephone or video call.  Interview duration ranged from 54 to 86 min.

### 2.5 Measures

The semi‐structured *Temper Outburst Interview* schedule (TOI; Tunnicliffe et al., 2014) comprised 32 questions intended to elicit a phenomenological account of temper outbursts from a caregiver perspective. It included open‐ended questions to encourage description of idiosyncratic behaviours. Questions covered the latency and duration of outbursts; common antecedents; precursor behaviours; type and sequence of behaviours during a typical outburst; and the success or otherwise of management strategies used by caregivers to alleviate harm or reduce outbursts. Coding instructions for each question were taken from the study by Tunnicliffe et al. (2014) enabling quantitative analysis and comparisons with descriptions of outbursts in PWS reported by Tunnicliffe et al. (A copy of the interview schedule can be made available on written request to the corresponding author).

The interview was previously shown to have good convergent validity with parental diary records of temper outbursts in PWS (66%‐100%; Tunnicliffe et al., 2014). In order to reduce research burden on informants, a decision was taken not to include a diary study as part of the present research. A proportion of the questions were taken directly from the Challenging Behaviour Interview, which has established reliability (Oliver et al., 2003; inter‐rater reliability: 0.69, test–retest reliability: Pearson's *r* = 0.90).  Five of the interviews were coded independently by two researchers to assess inter‐rater reliability. This was calculated as the percentage agreement on each question of the interview schedule. Agreement ranged between 60% and 100%, overall agreement was 85%. Fourteen of 30 questions had 100% agreement.

### 2.6 Coding and data analysis

For data analysis purposes, information from the joint interview was combined and treated as for a single informant. Where differences existed in initial response a consensus was agreed between the two informants. All behaviours noticed by either informant were included in the descriptive account.

To reduce descriptors of specific behaviours to a manageable number and allow comparison, behaviours were grouped into categories (Table 2). Avoiding direct replication of the categories used by Tunnicliffe et al. (2014) allowed for the emergence of additional behaviours applicable to LS.  Setting events, which increase the likelihood of an outburst, were categorized into physiological, environmental and social factors according to McGill, (1999).

**Table 2:** Categories of behavioural topographies

Categories	Behaviours included
Perseverative requests	Repetitive questions, or continuing requests for an item or object, or requests to avoid unwanted activity
Non‐compliance	Refusal to comply with request, for example, to use bathroom, put shoes on
Facial expression	Angry facial expression, “screwing up his face,” grimacing, scowling
Physiological arousal	Red face, sweating, panting (as if out of breath)
Increased motor activity	Pacing, rocking, hand‐flapping, twisting fingers, flailing arms and legs, stamping feet, biting or twisting tongue, gritting or clenching teeth
Dropping	Throwing self to the floor from a seated or standing position, throwing body back in wheelchair
Talking	Talking to self, talking to other
Self‐deprecating speech	“I'm so stupid,” “I'm no good”
Verbal aggression	Verbal threats, insults, swearing at others, argumentative
Emotional vocalizations	Shouting, yelling, screaming, squealing, growling, saying “I'm scared.”
Crying	Sobbing, tearful
Self‐injury	Hitting self, hand‐biting, pulling or twisting body parts, hitting self against furniture or hard surfaces
Physical aggression (towards others)	Hitting, kicking, biting, scratching, pinching, digging nails into skin (drawing blood), headbutting, hairpulling
Aggression towards property	Hitting or kicking walls, windows, floors, slamming doors, overturning furniture, throwing objects
Antisocial acts	Spitting, deliberate defaecation, urination, rectal digging, smearing
Destructive	Tearing, ripping objects, or spoiling an activity (e.g., overturning a game, taking toys from others.)
Avoidance behaviour	Walking away, ask to go to hallway, go to porch, go to bedroom
Resumes activity	Sudden return to a calm state, goes back to what they were doing before the outburst “as if nothing has happened”
Relationship repair	Apologizes, says sorry, asks for a cuddle, asks “mummy happy?”, loving, kissing, hugging, makes tea for mother
Exhausted	Tired, lies down, goes to sleep
Other	Goes for a walk to self‐soothe, has a shower to wash away bad feeling, lies down or falls asleep

Data were analysed using Pearson's chi‐squared test for comparisons with data on PWS from Tunnicliffe et al. (2014). Fisher's exact tests were used to verify results where appropriate. Given the clinical importance of the study and the rarity of the syndrome leading to a relatively small number of participants, a Bonferroni correction (*p* < 0.002) was considered too conservative hence an Alpha level of *p* < 0.01 was adopted.

## 3 RESULTS

### 3.1 Latency and duration of temper outbursts

Data on the latency and duration of outbursts are shown in Table 3. Temper outbursts were a frequent occurrence for all 17 participants. Latency data indicated that 14 out of 17 participants experienced outbursts at least once a day with 8 informants reporting expected outbursts within the next hour. Typically outbursts lasted less than 15 min for 12 out of 17 participants. Of the three informants reporting longer latency periods two reported weekly occurrence of outbursts lasting between 5 and 15 min, and one would only expect to see the next outburst within a month and typically lasting less than 5 min. Of those reporting durations of between 15 and 60 min, one reported a latency of fifteen minutes, one expected an outburst within the next hour, and one reported outbursts of between 30 and 60 min as a daily occurrence. One informant experienced daily outbursts of more than one hour, with a maximum duration of 3 hr.

**Table 3:** Latency and duration of temper outbursts

Response	Frequency *N* = 17
Latency to the next outburst:	
Within the next 15 min	2
Within the next hour	6
By this time tomorrow	6
By this time next week	2
By this time next month	1
Duration of longest outburst in the last month:	
Less than a minute	0
Less than 5 min	4
Less than 15 min	4
Less than an hour	5
More than an hour	4
Duration of typical outburst:	
Less than a minute	2
Less than 5 min	5
Less than 15 min	5
Less than an hour	4
More than an hour	1
Length of the longest outburst over one hour (minutes)	*N* = 7
90 min	1
120 min	1
180 min	3
240 min	2

Informants were asked to identify factors likely to lead to prolonged outbursts. Saying “no,” “not getting his own way,” or “being forced to do something he did not want to do” were cited by eight informants as the main reason for extended outbursts. “Frustration” was identified by three informants, anxiety by two and ignoring or not paying sufficient attention by a further four. Obsessive behaviours and an inability to “let go of an issue” were mentioned by two informants.

### 3.2 Setting events

Table 4 provides a list of the setting events identified. Physiological or internal setting events, included tiredness (*n* = 7), hunger (*n* = 4), anxiety/fear (*n* = 5) and physical pain or discomfort (*n* = 5). Low mood (*n = *1) and thirst (*n* = 1) were also mentioned. Environmental factors included time pressure (*n* = 2), or generalized change to routine such as being on holiday (*n* = 5) or being in unfamiliar surroundings (*n* = 3). Many informants noted that high ambient or unexpected noise levels (*n* = 9) or crowded situations (*n* = 4) increased the likelihood that an outburst would be triggered.

**Table 4:** Physiological, environmental and social setting events

Setting event	*N* ^ª^
Physiological (any of the below list)	12
Tiredness	7
Hunger	4
Thirst	1
Low mood	1
Anxiety/fear	5
Physical pain or discomfort	5
Environmental (any of the below list)	17
Time pressure	2
General change to routine (e.g., holidays)	5
Coming home	1
Unfamiliar setting	3
Crowds	4
Noise levels high or unexpected	9
Social (any of the below list)	**7**
When with certain person	5
Relationship difficulties	5
Embarrassment	1

^ª^ Some informants reported more than one setting event within each category.

### 3.3 Antecedents

Table 5 provides information about the principal antecedent identified by informants. Two informants said that they could not identify a trigger for the specific episode described but were able to report the most common trigger for outbursts in general. Nine out of 17 informants indicated that some form of thwarted desire was the most prevalent trigger for an outburst. This included frustrated goals (*n* = 1), delayed gratification (*n* = 1), “not getting what he wants” or “not getting his own way” (*n* = 6), “not being able to do something he wants to do” (*n* = 1). Two other informants stated that “being asked to do something he does not want to do” leads to most outbursts. Change to routine or uncertainty about expectations provoked regular outbursts for three participants. Two informants noted that unexpected change in auditory stimulation such as a car engine stopping, or the TV or radio switching to advertising, triggered outbursts. One informant identified boredom or frustration as the main trigger. There was no robust evidence of an association between the principal antecedent and individual characteristics such as chronological or developmental age or additional diagnoses.

**Table 5:** Principal antecedents to each participant's temper outbursts

Part. Ref.	AEª	Principal antecedents	Proportion of all temper outbursts preceded by principal antecedent	Does antecedent always lead to an outburst?	What is different on occasions when antecedent does not lead to an outburst?
1	3:3	Not getting what he wants	9/10	Yes	*N*/a
2	4:3	Not getting what he wants	9/10	Yes	Sometimes willing to negotiate
3	4:11	Not getting what he wants	7/10	No	Environment—no outbursts at school. People—usually with mother or brothers, less often with father
4	2:6	Delayed gratification	10/10	Yes	People—having father around Environment—no outbursts in school or respite
5	3:3	Not getting his own way	8/10	No	Environment—no outbursts in school or public Parents more likely to negotiate in public
6	4:6	Wanting something and being tired	9/10	No	Environment—no outbursts in school or public
7	5:3	Not getting what he wants	8/10	No	People
8	2:2	Something stopping (e.g., TV, radio, car engine)	5/10	Yes	Environment—no outbursts at school. People—more with mother than father. Gradual reduction in noise?
9	1:8	Noise (e.g., from kitchen), TV or radio going to commercial.	7/10	No	Not clear—possibly volume, or mood
10	0:10	Boredom or frustration	8/10	Yes	*N*/a
11	10:8	Change to routine	8/10	No	Environment—no outbursts in public
12	10:6	Not being able to do something he wants to do	9.5/10	Yes	*N*/a
13	8:11	Frustrated goals	8/10	No	How decision is presented, negotiation
14	5:11	Doing something he does not want to do	7/10	Yes	If he wants to go somewhere
15	1:9	Uncertainty	9/10	No	People—different carers, better with father
16	0:10	Being asked to do something he does not want to do	9/10	No	Physical discomfort
17	–	Change in routine or expectation	8/10	No	Catch it quickly and acknowledge mistake

^ª^ AE = age equivalent score in years: months, from VABS‐II scores (Sparrow et al., 2005), see Table 1.

**Figure 1:**
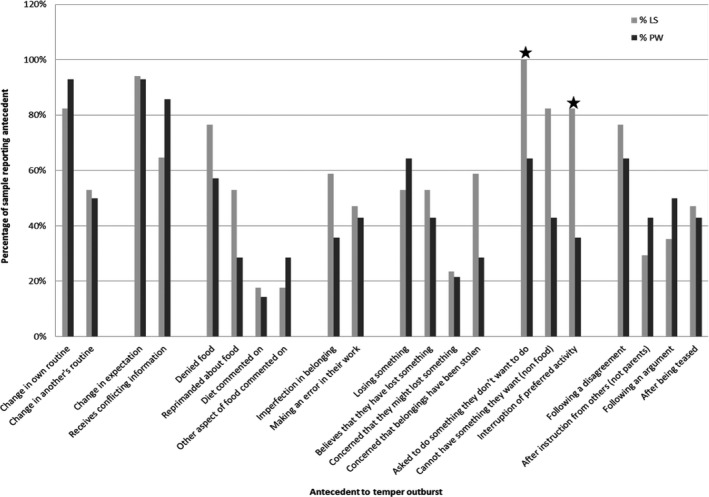
Percentage of informants reporting antecedent to temper outburst in preceding twelve months

All informants reported multiple potential triggers for outbursts, ranging from five to 18 out of 21 possible antecedents suggested. The results are presented in Figure 1 together with the results from Tunnicliffe et al. (2014) for parents/carers of people with PWS. The graph shows that all LS informants (*n* = 17; 100%1Although small numbers (*n < *20) would normally preclude use of percentages, they are shown here and throughout the paper where inclusion aids comparison with results from Tunnicliffe et al. (2014).) reported witnessing a temper outburst triggered by the participant being asked to do something they did not want to do.  The next most commonly reported antecedents in LS were change in expectation (*n* = 16; 94%), change in own routine (*n* = 14; 82%), not getting something they want (*n* = 14; 82%) and interruption to preferred activities (*n* = 14; 82%). Denial of food and disagreements were both reported in 76% (*n* = 13) of LS participants, and imperfections and concerns that belongings had been stolen were reported in 59% (*n* = 10) of participants. These results are consistent with the individual antecedents reported above.

### 3.4 Sequence of behaviours during an outburst

**Figure 2:**
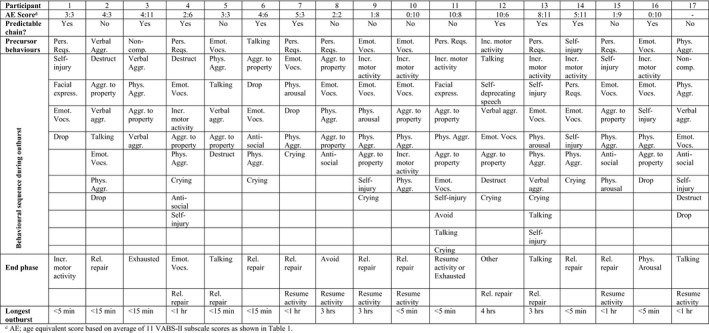
Sequence of behaviours shown by each participant during temper outburst

Individual behavioural sequences, using coded topographies from Table 2, are shown in Figure 2.  These are based on description of the last severe outburst (as defined by informant) observed during the month preceding the interview. A predictable pattern of behaviours during outbursts was shown by 9/17 participants. The small sample size did not allow for identification of statistically significant associations.

All informants were able to identify precursor behaviours which alerted them to a potential outburst but preceded the point at which an outburst could no longer be avoided. Seven informants reported perseverative requests, and four mentioned emotional vocalizations as a warning sign (e.g., shouting or yelling), differentiated from the start of an outburst by a clear change in tone or volume. Other precursors included: self‐injury, verbal or physical aggression towards others, non‐compliance with requests, increased motor activity or talking to self (verbalizing thoughts of displeasure).

The most common behaviours during outbursts were emotional vocalizations (*n* = 15) and physical aggression (*n* = 15). Aggression to property such as kicking or hitting walls or throwing objects was reported by 12 informants, and verbal aggression (e.g., swearing or shouting directed at another person) was reported in six cases. Of those showing externally directed aggression, 14 showed multiple forms of aggression, with seven displaying physical aggression towards others and towards property and four displaying verbal and physical aggression towards people and property. Of the nine participants exhibiting self‐injury all except one also showed physical aggression towards others. Crying (8/17), which is distinguished from other emotional vocalizations, was reported in the middle and towards the end of outbursts. This contrasts with reported crying behaviour in PWS (Tunnicliffe et al., 2014) which occurred at the start and end of outbursts but never in the middle.

Behaviours during the end phase of an outburst showed two distinctive patterns. Eleven of the seventeen informants reported relationship repair behaviours including apologizing, asking for a hug or seeking reassurance from caregivers. For six of those eleven, crying or dropping (important indicators of distress according to Potegal & Davidson, 2003) immediately preceded attempts to repair relationships with caregivers. Seven informants reported that the participant would suddenly go back to their previous activity and emotional state as if nothing had happened, but in four of those cases this only occurred after reassurance had been provided by caregivers. Attempts to self‐soothe were also present, with self‐talk reported by three informants.

Specific behaviours coded as “antisocial” were identified for five participants. These behaviours included spitting (*n* = 2) and deliberate urination, defaecation and smearing (*n* = 4). All five of these individuals also demonstrated physical aggression towards others, and either aggression towards property (*n* = 4) or verbal aggression (*n* = 1). Three of these participants also exhibited self‐injurious behaviours. There was insufficient evidence to suggest an association between this behaviour and participant characteristics such as age or developmental abilities.

The most frequently reported perceived emotions during an outburst were frustration (*n* = 12) and anger (*n* = 8).  These sometimes occurred together.

### 3.5 Management strategies used by caregivers

At the precursor stage, seven informants reported that the most successful strategy was distraction or redirection to an alternative activity. Other strategies included calm reasoning (*n* = 2), removal of choice (*n* = 1), providing attention (*n* = 1), offering help (*n* = 1), reiterating clear routine (*n* = 1), removing other children from the room (*n* = 1) or giving in (*n* = 1).  Informants estimated that success rates for avoidance of an outburst were between 40% and 90% at this stage.  Only one informant felt that there was nothing that could be done even at the precursor stage. Table 6 gives a list of principal strategies and the success rate for each.

**Table 6:** Principal strategies and success rates

Preventative strategy at precursor stage	*N*	Success rate
Discussion/calm reasoning/negotiation	3	60%–80%
Distraction/redirection (incl. use of humour)	7	50%–90%
Consequences (e.g., removal of tangible or aversive consequence)	1	80%
Provide attention/offer help	2	40%–80%
Give in to demands	4	70%–100%
Withdraw person with Lowe syndrome from situation	2	90%
Nothing works	1	0%
Principal strategies during outburst		
Discussion/calm reasoning/negotiation	4	0%–60%
Distraction/redirection (incl. use of humour)	3	0%–50%
Consequences (e.g., removal of tangible or aversive consequence)	3	0%–60%
Ignore/withdraw attention	3	0%
Withdraw person with Lowe syndrome from situation	3	0%–60%
Restraint	2	Harm reductionª

Other strategies described by individual informants		
Shouting	1	Not reported
Yelling “stop”	1	Not reported
Singing to him	1	Not reported
Provide choice	2	Not reported
Limit choice	1	Not reported

^ª^ 0% success in stopping outburst but used to prevent physical harm to self, carer, other person or property.

During an outburst, the chances of successful intervention reduced and the main aim of intervention at this stage appeared to be harm reduction, either to the person with LS, others at risk of aggression, or to avoid damage to property. Removal of the participant to a quiet location or withdrawal of the caregiver avoided further escalation but did not immediately stop an outburst. Redirection, humour or distraction was reported to be successful in 60%–90% of outbursts if the intervention was made early enough.

The most common reason for variation in intervention strategies was location (*n* = 10). Concern for the judgement of others and risk to others’ safety when in public were given as reasons for variation. Informants also reported that they would be more likely to intervene directly rather than ignore behaviour, or might withdraw for their own safety, when the participant became aggressive.

### 3.6 Comparison with Prader–Willi syndrome

Data were extracted from the Tunnicliffe et al. paper, and the two samples compared to check for differences in mean age or adaptive abilities (Tunnicliffe et al., 2014). No significant differences were found in VABS‐II adaptive behaviour composite scores (Mann–Whitney *U*, *p* = 0.377), but a difference was found in the chronological age profiles of the two samples (*t* (29) = −1.44; *p* = 0.018), with a higher mean age in years reported for PWS. When a comparison was made based on age group (<18 years; ≥18 years) no significant difference was found between the two groups (*p *> 0.05). An important difference between the two samples is that participants with PWS were selected on the basis that routine change was a trigger for outbursts. This was not a requirement for the LS participants due to the rarity of the disorder but should be taken into consideration when interpreting the data.

The following differences were noted between the two samples. Crying (Fisher's exact, *p* = 0.008) and running away (Fisher's exact, *p* = 0.010) were more frequently reported in the PWS group. Physical aggression towards others was more frequently seen in LS (Fisher's exact, *p* = 0.010). Antisocial acts (spitting, deliberate defaecation or urination or smearing) were not reported at all in descriptions of outbursts in PWS but were reported by five informants in the LS study.  This difference only approached statistical significance (Fisher's exact, *p* = 0.036).

There was no significant difference in outbursts occurring in response to routine changes. The similarity in the prevalence of routine change as an antecedent enables other comparisons to be made despite this difference in initial selection criteria. Differences in the pattern of antecedents reported during the last twelve months were significant at *p* < 0.01 for “asked to do something they don't want to do” (ᵡ^2^ = 7.24; *p* = 0.007) and for “interruption of preferred activity” (ᵡ^2^ = 7.04; *p* = 0.008). Both these factors were reported more frequently in LS than in PWS. The sudden resumption of activities as if nothing had happened was not reported at all in PWS but was spontaneously mentioned by eight informants in the LS study (Fisher's exact, *p* = 0.003).  No other significant differences were found.

## 4 DISCUSSION

Temper outbursts have previously been shown to be more prevalent in LS than in other people with intellectual disabilities or in typical development (Dolinsky et al., 2008; Kenworthy & Charnas, 1995). The primary aim of this investigation was to generate a description of temper outbursts in LS based on informant accounts.  Seventeen interviews with eighteen caregivers provided detailed accounts of the antecedents, behavioural and emotional sequence, and the consequences of temper outbursts in 17 people with LS.

It is notable that all participants with LS were eight years or older, putting them above the expected chronological age of five years for reduction or cessation of temper outbursts in typically developing children (Potegal & Davidson, 2003). Developmental age, however, as measured using age equivalent scores from the VABS‐II, showed that more than half the participants had a developmental age of below five years.  The topographies of behaviour during outbursts in LS bear marked similarities to those described for temper outbursts in typically developing children aged 2–5 years (Österman & Björkqvist, 2010; Potegal & Davidson, 2003), and “angry‐agitated outbursts” in paediatric inpatients (Carlson et al., 2009; Potegal, Carlson, Margulies, Gutkovitch, & Wall, 2009).

In pre‐school children, Wakschlag et al., (2007) suggested that both quality of behaviours (severity) and pervasiveness (frequency and duration) should be considered when determining the degree of pathological emotional dysregulation.  In the current study of people with LS, most informants reported outbursts as a daily occurrence and nearly half reported a latency of an hour or less.  Durations varied between less than five minutes and over an hour, compared with 0.5 to 40 min (*M = *3* *min*)* previously reported in typically developing children (Potegal, Kosorok, & Davidson, 1996).  The prevalence of physical and verbal aggression towards others and towards property is indicative of a high level of severity, with implications for the well‐being of both carers and people living with LS.

The reason for occurrence of distressing behaviours such as smearing, deliberate defaecation or urination and spitting is unclear. One hypothesis proposed by several informants in this study was the difficulty carers had in disregarding such behaviours. They reported feeling obliged to respond, particularly when the behaviours impacted on others. This also applied to extreme aggression towards carers, attacks on siblings or strangers, or dangerous behaviours such as kicking windows. This hypothesis would be consistent with a functional behavioural analysis of challenging behaviours being inadvertently reinforced by attention, escape from demands or distraction with tangible reward (Iwata et al., 1994; Warren & Mondy, 1971) although further research is needed to understand the functions of these behaviours.

When exploring the aetiology of temper outbursts in genetic syndromes it is important to consider the role of physical differences. LS is characterized by significant physical impairment (Lewis et al., 2012) with associated limitations to independent access to tangible items, and the possibility of physical pain and discomfort. Frustration was cited as both a triggering event and a reason for prolonged outbursts. Informants sometimes perceived this as resulting from inability to perform a physical task to a desired standard, or without physical assistance from a caregiver (e.g., toileting) or lack of independent choice over timing or content of activities.

Physiological setting events were commonly identified as increasing the likelihood of an outburst, including hunger, thirst and tiredness. It is also interesting to note the environmental factors which impact on outbursts. Change in ambient noise or sudden changes in auditory stimuli were reported by more than half the respondents as increasing the likelihood of an outburst. Increased sensitivity to noise (hyperacusis) has been noted as a feature of other genetic disorders such as Cri‐du‐chat, and Williams syndromes but was not previously found to be associated with LS (Cornish & Pigram, 1996). Increased physiological arousal or anxiety caused by unusual sensitivity to sensory stimuli have been noted as a potential contributory factor in challenging behaviour in other disorders such as autistic spectrum disorders (ASD; Grapel, Cicchetti, & Volkmar, 2015) and Williams syndrome (John & Mervis, 2010). One of the participants for whom change in ambient noise was the principal antecedent for outbursts had a comorbid diagnosis of ASD but the other did not have this diagnosis. Another interesting aspect of environmental setting is the reported absence of temper outbursts outside the home, and a difference in behaviours dependent on who the carer is (e.g., mother or father). The “context‐specificity” of outbursts may offer scope for environmental interventions to reduce the frequency or intensity of outbursts but further research would be needed to understand why self‐regulation is possible in some circumstances but not in others.

This study has highlighted the potential importance of frustration intolerance as a factor in temper outbursts in LS. The current research did not use functional analysis methodology but the fact that all informants reported outbursts in response to unwanted demands suggests that escape may be a prominent driver of outbursts for this population. Further functional analytical research would be required and comparison with other intellectual disability populations to establish whether this is true.

The absence of difference between LS and PWS populations in reports of routine change as an antecedent is interesting given that the PWS group were selected on this basis, and the LS group were not. Change to routine has been noted as a potential trigger for temper outbursts in a number of genetic syndromes including PWS, LS, fragile X and Smith Magenis syndromes (Bull, Oliver, & Woodcock, 2017).  A link has also been made between intolerance of change and repetitive behaviour as a precursor to outbursts (Moss, Oliver, Arron, Burbridge, & Berg, 2009).  In the current study, perseverative requests were frequently reported as a precursor and change to routine or expectation was reported as antecedent to temper outbursts.

More than half of respondents spontaneously identified some form of thwarted goal‐directed behaviour as the principal antecedent for outbursts. Thwarted goal‐directed behaviour differs conceptually from traditional functional behavioural analysis. It incorporates elements of all three operant conditions including demands generated by the individual (i.e., access to attention or tangible reward) and desire to escape from an unwanted demand or unpleasant stimulus (such as high ambient noise). This concept also appears to be supported by findings from a recently published study by Rice, Woodcock, and Einfeld (2018) who found that “goal blockage” was one of three key factors likely to provoke outbursts in people with PWS. It suggests an alternative to a functional behavioural model for understanding temper outburst behaviours which acknowledges the importance of internal emotional state and the development of inhibitory control mechanisms for management of external emotional expression.

Österman and Björkqvist (2010) described tantrums in typical development as a response to frustrated desire. They noted that the most rapid decline in outbursts occurs at the age of around four years when children start to develop more sophisticated language to express their emotions, including anger and frustration. It also coincides with the development of other social skills which enables a person to get their needs met. In this study, there was no significant association between the communicative or social abilities of participants and the latency or duration of outbursts, but the small sample size may have led to a type II error and finding no association where one might conceivably exist.

The inability to tolerate frustration in young typically developing children and in older paediatric psychiatric inpatients is thought to be due to underdevelopment of cognitive mechanisms which control and regulate emotions and behaviour, known as executive functions (Hunter & Sparrow, 2012). Executive functions cover a range of cognitive abilities including judgement, planning, impulsivity, behavioural inhibition and task switching. It is of interest that some participants reported that their children showed remorse or relationship repair following an outburst. This may suggest that, at least for a subset of individuals with LS, temper outbursts were experienced as a loss of control over emotions, which later gave rise to the motivation to apologize or express remorse.

Executive function deficits may be implicated in temper outbursts in a range of genetic syndromes. For example, in PWS, a strong association has been found between task switching deficits, change to routine and temper outbursts (Woodcock et al., 2010). The cognitive challenge of moving from a well‐rehearsed sequence of behaviour to adapt to a new task is thought to be aversive for individuals with PWS, which then overwhelms emotional coping skills. The discovery of task switching difficulties in PWS has led to the development of promising interventions to support transitions between activities and reduce the incidence of outbursts (Bull et al., 2017). In addition, recent research has indicated that vagal nerve stimulation leads to improvements in behavioural difficulties in PWS, supporting a view that there is heightened propensity to emotional reactivity in PWS due to dysregulation of the autonomic nervous system, and that vagal nerve stimulation modulates projections to various limbic and forebrain cortical areas (Beresford‐Webb, Manning, Aman, & Ring, 2018; Manning et al., 2016). This research further indicates the importance of understanding neurological difference in behavioural aetiology.

Recent MRI studies have shown non‐specific abnormalities in brain development of people with LS, including delayed myelination and the presence of small cystic lesions in the white matter (Allmendinger, Desai, Burke, Viswanadhan, & Prabhu, 2014). The implications of these findings are not yet clear but a better understanding of neurological functioning in people with LS could be an important next step in developing effective interventions for management of temper outbursts in this group.

### 4.1 Limitations of the study

The TOI semi‐structured interview schedule included open‐ended questions to allow for the emergence of a detailed descriptive account of temper outbursts in LS. It provided structure for the collection of frequency data for comparison with behaviours in other populations. The schedule had been written however specifically for research on PWS and may therefore have overemphasized factors important to that population. A large‐sample questionnaire study by Rice et al. (2018), published since the current study, reports that over 90% of respondents with PWS have had outbursts within the last year provoked by “being told no” or “being asked to do something they do not want to do.” This is higher than the percentages reported in Tunnicliffe at al. (2014) used for comparison in the current study. This suggests that the differences between PWS and LS reported within the current study may have arisen because of specific selection criteria in the Tunnicliffe et al. paper, the use of interview schedules as opposed to questionnaires, or because of small sample sizes. Importantly, cross‐syndrome comparisons of LS and PWS using consistent methodology are needed in the future to further delineate the similarities and differences between these groups.

Reliance on informant report gives limited insight into the perspective of the person with LS, which is a major flaw in the methodology. In future, it would be important to devise methods for enabling those with limited communication skills to contribute to understanding of outburst behaviours and provide insight into internal emotional state before, during and after an episode. Informant report is also potentially problematic as caregivers will be influenced by their own attributions about the cause of behaviours that challenge, available social discourse on temper outbursts in typical development and their own levels of stress tolerance.

Future research could include objective observation of temper outburst behaviours in an experimental or naturalistic setting to provide additional scientific rigour and more accurate measurement and description of the frequency, duration, severity and sequence of component behaviours to complement descriptive accounts. Statistical comparisons have been made with findings from Tunnicliffe et al., 2014 in PWS but should be treated with caution as the number of participants in each of these studies is small.

## 5 CONCLUSIONS

This is the first paper to provide a robust descriptive analysis of temper outbursts in LS. Plausible hypotheses have been generated based on parental attributions of cause and comparisons with outbursts in other populations.  Note has been made of the high prevalence of aggression in outbursts in LS and the frequency of thwarted goal‐directed behaviour as a possible trigger.

One of the important aims of investigating challenging behaviours in genetic disorders is to develop effective preventative and management interventions to reduce distress for the individual and their carers. Further research is needed to determine whether under‐developed emotion regulation or other cognitive mechanisms may be contributing to the frequency and severity of outbursts in LS. Evidence is emerging from ongoing research that suggests that this is the case.  Depending on the outcome of such research, successful interventions could be developed to strengthen emotional control or to reduce the cognitive challenge of particular situations or tasks. Promising research has been developed for managing task switching deficits in PWS (Bull et al., 2017) and in the use of effective parenting techniques to teach emotional recognition and control to preverbal typically developing children (e.g., Douglas, 2007).  With better understanding of the gene‐environment‐behaviour pathway (Tunnicliffe & Oliver, 2011), it is possible that these techniques could be adapted for children with LS and other syndromes in which temper outbursts are frequent.  This paper has therefore made an important contribution to the literature on temper outbursts in intellectual disability populations and may have moved us closer to understanding the complex interplay between emotion, behaviour regulation and neurological difference in genetic syndromes.
